# Risk factors associated with overall survival in patients with multiple myeloma following carfilzomib treatment: A retrospective study from a large claims database in Japan

**DOI:** 10.1002/cam4.6457

**Published:** 2023-09-26

**Authors:** Hiromi Hagiwara, Takafumi Nakayama, Hiroya Hashimoto, Shigeru Kusumoto, Hidekatsu Fukuta, Takeshi Kamiya, Koichi Ikuta, Shinsuke Iida

**Affiliations:** ^1^ Department of Medical Innovation Nagoya City University Graduate School of Medical Sciences Nagoya Japan; ^2^ Department of Cardiology Nagoya City University Graduate School of Medical Sciences Nagoya Japan; ^3^ Department of Clinical Research Management Center Nagoya City University Hospital Nagoya Japan; ^4^ Department of Hematology and Oncology Nagoya City University Graduate School of Medical Sciences Nagoya Japan; ^5^ Laboratory of Immune Regulation, Department of Virus Research, Institute for Life and Medical Sciences Kyoto University Kyoto Japan

**Keywords:** carfilzomib, large claims database, lenalidomide, multiple myeloma, overall survival, renal impairment

## Abstract

**Background:**

Carfilzomib is a selective proteasome inhibitor approved for treating relapsed or refractory multiple myeloma (RRMM). Carfilzomib improves overall survival (OS) and progression‐free survival (PFS); however, treatment with carfilzomib results in a higher incidence of cardiovascular and renal toxicity. More than 70% of patients with RRMM in clinical practice do not meet the eligibility criteria for randomized clinical trials (RCT). OS and PFS are negatively influenced by complications, concomitant medications and prior treatments. Therefore, we assessed the risk factors influencing the OS and time to next treatment (TTNT) in the real world. TTNT has emerged as a relevant alternative clinical endpoint to PFS.

**Methods:**

A retrospective analysis of a large claims database prepared during the post‐marketing stages in Japan was performed. The patients treated with carfilzomib for the first time were identified. Multivariable Cox proportional hazards regression analysis was performed to evaluate the risk factors influencing OS and TTNT following carfilzomib treatment.

**Results:**

A total of 732 patients with RRMM who received carfilzomib‐containing chemotherapy between April 2014 and September 2021 were identified. Multivariable Cox regression analysis for OS and TTNT showed a significantly higher hazard ratio (HR) of 1.48 (95% confidence interval [Cl]: 1.10–2.00; *p* = 0.010) and 1.38 (95% Cl: 1.15–1.65; *p* < 0.001), respectively, for patients with renal impairment compared to those without renal impairment. Multivariable Cox regression analysis for OS and TTNT showed a significantly higher HR of 1.80 (95% Cl: 1.27–2.55; *p* = 0.0010) and 1.38 (95% Cl: 1.14–1.66; *p* < 0.001), respectively, for patients with prior lenalidomide treatment compared to those without prior lenalidomide treatment.

**Conclusion:**

Complication of renal impairment and prior lenalidomide treatment could be risk factors influencing OS and TTNT during carfilzomib treatment.

## Graphical abstract

This retrospective observational study investigated the long‐term safety of anticancer drug carfilzomib which has cardiotoxicity in the real world. More than 70% of the multiple myeloma patients did not meet the eligibility criteria for the randomized clinical trials conducted for carfilzomib approval. Therefore, it is important to assess the long‐term safety from the perspective of clinical pharmacology and onco‐cardiology.

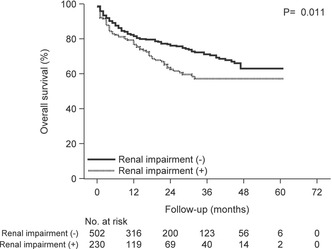

## 1 INTRODUCTION

Multiple myeloma (MM) is the third most common hematologic malignancy in Japan. The development of proteasome inhibitors (PIs) has improved progression‐free survival (PFS) and overall survival (OS) in patients with MM.^1^ Carfilzomib is a selective proteasome inhibitor that irreversibly binds to the beta 5 subunit of the proteasome, eliciting anti‐myeloma activity through unfolded protein stress response and other mechanisms.^1^

Carfilzomib is associated with cardiac and renal toxicities.^2^, ^3^, ^4^, ^5^ According to a recent study, renal failure was reported in 22% of cases treated with carfilzomib.^5^ However, phase 3 trials with regimens including carfilzomib, such as ASPIRE, A.R.R.O.W., ENDEAVOR and CANDOR, excluded patients with renal impairment and hypertension.^1^, ^6^, ^7^, ^8^, ^9^, ^10^ Several randomized clinical trials (RCTs) have evaluated the efficacy of carfilzomib. However, a discrepancy between the RCTs and routine clinical practices concerning OS and PFS was assumed because the RCT inclusion criteria were not uniform.

The previous study showed that up to 72.3% of patients in routine care did not meet the eligibility criteria of the RCTs for approval.^5^ OS was significantly worse (~50% increased risk of mortality) in patients with relapsed or refractory MM (RRMM); this resulted in an inability to meet the trial eligibility criteria. OS was negatively affected by the inability to meet the RCT eligibility criteria, highlighting the lack of generalizability of RCT results to the real‐world RRMM population.^5^, ^11^

Therefore, it is essential to clarify the benefits and risks associated with carfilzomib treatment. There is a need to understand the effects of risk factors such as comorbidity and concomitant medications, which were used as exclusion criteria in RCTs.

The present study aimed to explore the risk factors influencing the OS in the real world, using a large claims database. And we also examined the OS and time to next treatment (TTNT) following carfilzomib treatment by those risk factors. TTNT has emerged as a relevant alternative clinical endpoint to PFS.

The secondary purpose of this study was to play a role similar to post‐marketing surveillance. Recently, the use of real‐world data in drug development has increased, supporting regulatory decision‐making. The post‐marketing surveillance conducted by the company stated a follow‐up period of 6 months.^12^ That was too short to assess OS as a limitation. Therefore, we conducted a longer survey using real‐world data. To our knowledge, this is the first report of a longer follow‐up period of 7 years since the approval of carfilzomib in 2014.

## 2 METHODS

### 2.1 Study design and data sources

This is a retrospective study using a large claims database prepared during the post‐marketing stages in Japan. This database was constructed by JMDC; it includes monthly claims of 12 million patients from 323 medical institutions and pharmacies submitted between April 2014 and September 2021. The JMDC database provides information about the beneficiaries, including encrypted personal identifiers, age, sex, the International Classification of Diseases 10th revision (ICD‐10) diagnostic codes, and procedure codes, as well as the name of the prescribed or dispensed drugs for the inpatients and outpatients. We extracted the information on demographics, diagnoses, procedures, and concomitant medications from these data sets (Table S1).

### 2.2 Study population

The data of patients who received carfilzomib treatment for the first time between April 2014 and September 2021 were extracted. The index date was defined as the first month of carfilzomib treatment between April 2014 and September 2021.

### 2.3 Outcome and definition

**FIGURE 1:**
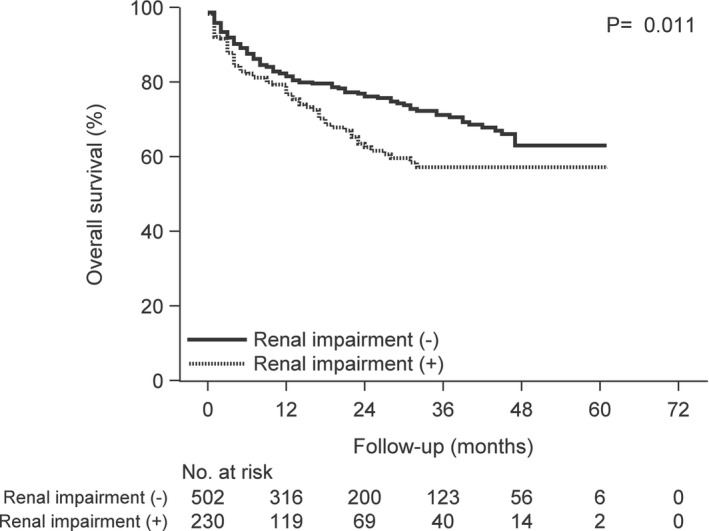
Kaplan–Meier curves of overall survival according to complication of renal impairment versus no renal impairment following carfilzomib treatment.

**FIGURE 2:**
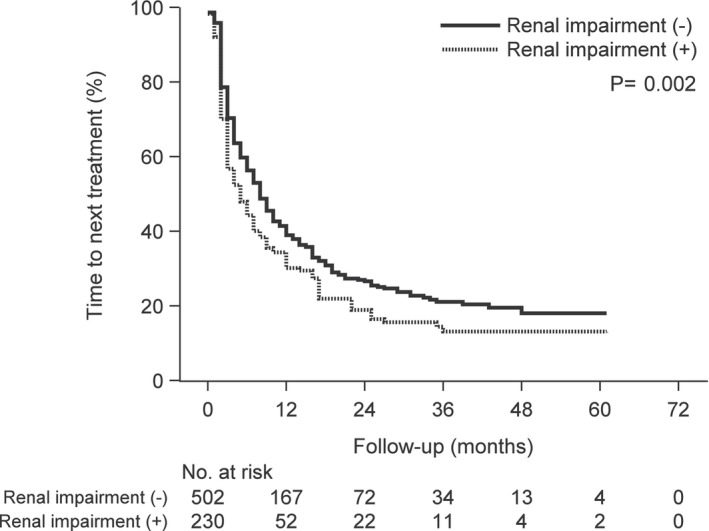
Kaplan–Meier curves of time to next treatment according to complication of renal impairment versus no renal impairment following carfilzomib treatment.

**FIGURE 3:**
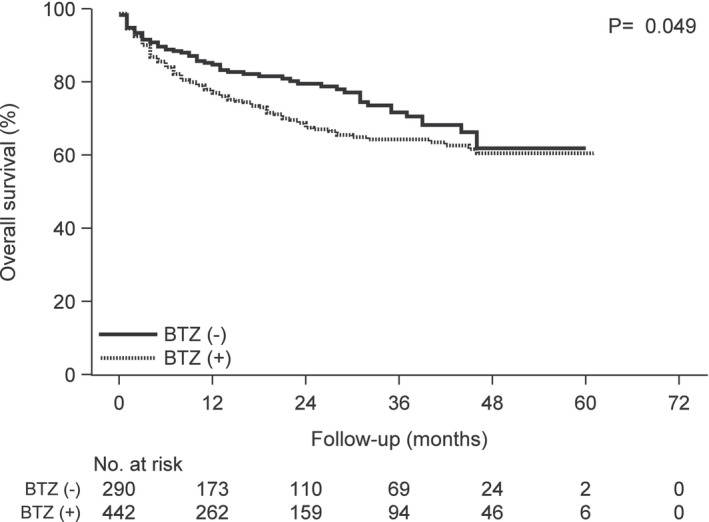
Kaplan–Meier curves of overall survival following carfilzomib treatment according to the prior bortezomib treatment group versus the group without prior bortezomib treatment. Abbreviation: BTZ, bortezomib.

**FIGURE 4:**
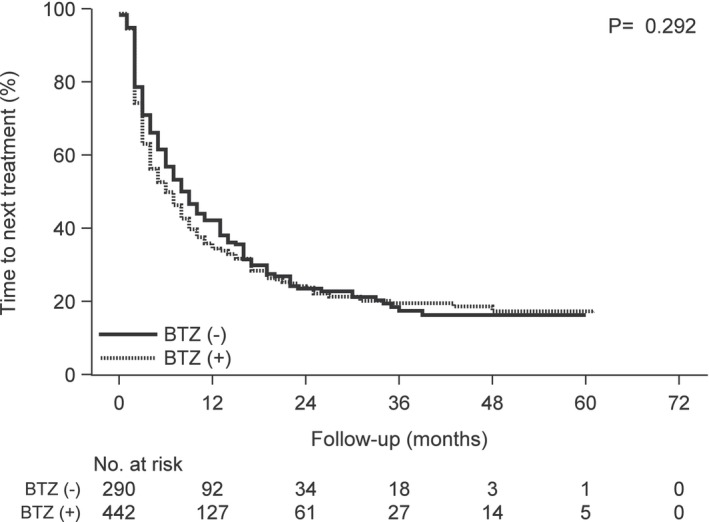
Kaplan–Meier curves of time to the next treatment following carfilzomib treatment according to the prior bortezomib treatment group versus the group without prior bortezomib treatment. Abbreviation: BTZ, bortezomib.

**FIGURE 5:**
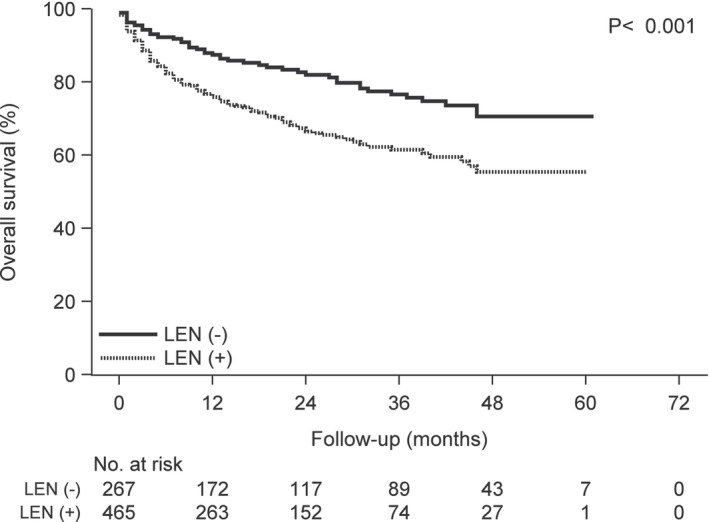
Kaplan–Meier curves of overall survival following carfilzomib treatment according to the prior lenalidomide treatment group versus the group without prior lenalidomide treatment. Abbreviation: LEN, lenalidomide.

**FIGURE 6:**
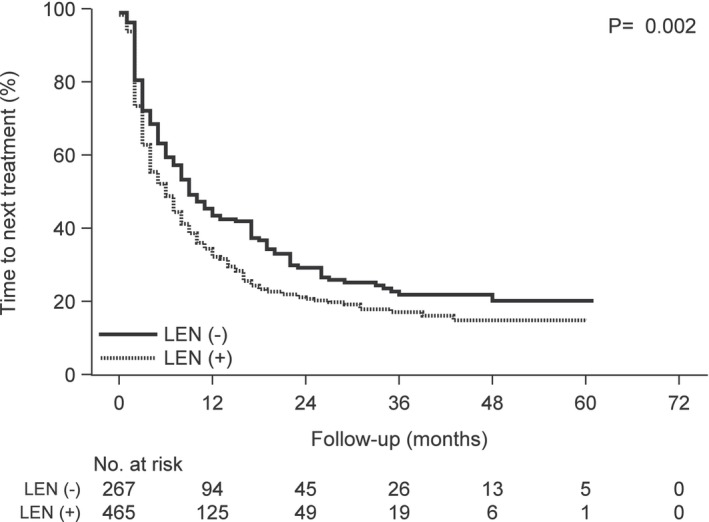
Kaplan–Meier curves of time to the next treatment following carfilzomib treatment according to the prior lenalidomide treatment group versus the group without prior lenalidomide treatment. Abbreviation: LEN, lenalidomide.

Detailed definitions of complications based on the ICD‐10 codes and concomitant medication are summarized in Table S1. The outcome was OS following initial treatment with carfilzomib‐containing therapies. TTNT was defined as the time from the index date to the first event, which was defined as the starting of the next treatment or death from any cause. For patients who did not experience the event, TTNT was defined as censored at the date of the last follow‐up. The next treatment was defined as the treatment with medication, which is summarized in Table S2. Renal impairment was diagnosed using the ICD‐10 code N17‐19 (acute kidney failure, chronic kidney disease, and unspecified kidney failure) upon initiation of carfilzomib‐containing chemotherapy in the same month and before the month prior to its start (Figures 1, 2, 3, 4, 5, 6; Tables 1, 2, 3, 4, 5). However, to differentiate between the diagnosis of renal impairment as a complication and as an adverse event of carfilzomib and to eliminate bias, we defined only cases in which renal impairment was diagnosed before the month prior to its start (Figures S1 and S2; Tables S3–S5).

**TABLE 1:** Baseline characteristics of carfilzomib treatment group.

	No. (%)	
	*n* = 732	
Characteristic	*n*	%
Age		
Median, range, (IQR), years	70.0, 32–90, (63–76)	
Sex		
Male	397	54
Complication		
Hypertension	272	37
Dyslipidemia	138	19
Diabetes mellitus	93	13
COPD	51	7
Ischemic heart disease	407	56
Valvular heart disease	57	8
Renal impairment	230	31
Atrial fibrillation and flutter	33	5
Concomitant medication*		
Statins	100	14
β‐blockers	42	6
NOAC/DOAC	65	9
CCB	239	33
ARB or ACEi	120	16
MRA	29	4
SGLT2	5	0.7
Metformin	24	3
Lenalidomide	417	57
Pomalidomide	106	14
Dexamethasone	724	99
Prior treatment		
Bortezomib	442	60
Lenalidomide	465	64

Abbreviations: ACEi, angiotensin converting enzyme inhibitor; ARB, angiotensin II receptor blocker; CCB, calcium channel blocker; COPD, chronic obstructive pulmonary disease; DOAC, direct oral anti coagulants; MRA, mineralocorticoid receptor antagonist; NOAC, novel oral anticoagulants; SGLT2, the sodium/glucose cotransporter 2 inhibitor.

* Concomitant medication was not always simultaneously given, but prescribed in the same month when carfilzomib was given.

**TABLE 2:** Results of multivariable Cox proportional hazards regression analysis for overall survival.

	Univariable			Multivariable		
	HR (95% Cl) *p*‐value			HR (95% Cl) *p*‐value		
Age						
≥75	1.39	(1.04–1.86)	0.025	1.25	(0.93–1.68)	0.14
Sex						
Male	0.98	(0.74–1.29)	0.87			
Complication						
Hypertension	1.11	(0.84–1.48)	0.46			
Dyslipidemia	0.86	(0.60–1.24)	0.41			
Diabetes mellitus	1.35	(0.92–1.99)	0.12	1.27	(0.86–1.86)	0.23
COPD	0.94	(0.52–1.70)	0.84			
Valvular heart disease	1.18	(0.70–2.00)	0.53			
Ischemic heart disease	1.05	(0.79–1.39)	0.75			
Renal impairment	1.46	(1.09–1.95)	0.011	1.48	(1.10–2.00)	0.010
Atrial fibrillation and flutter	1.60	(0.88–2.89)	0.12	1.30	(0.72–2.37)	0.39
Concomitant medication						
Statins	0.94	(0.63–1.40)	0.75			
β‐Blockers	1.17	(0.66–2.06)	0.59			
NOAC/DOAC	0.72	(0.43–1.20)	0.20			
CCB	1.14	(0.85–1.53)	0.37			
ARB or ACEi	0.93	(0.64–1.36)	0.70			
MRA	1.53	(0.80–2.91)	0.19	1.35	(0.69–2.66)	0.38
SGLT2	0.70	(0.09–5.67)	0.74			
Metformin	1.14	(0.48–2.73)	0.76			
Prior treatment						
Bortezomib	1.35	(1.00–1.81)	0.048	1.06	(0.76–1.47)	0.74
Lenalidomide	1.88	(1.37–2.58)	<0.0001	1.80	(1.27–2.55)	0.0010

Abbreviations: ACEi, angiotensin converting enzyme inhibitor; ARB, angiotensin II receptor blocker; CCB, calcium channel blocker; COPD, chronic obstructive pulmonary disease; DOAC, direct oral anti coagulants; MRA, mineralocorticoid receptor antagonist; NOAC, novel oral anticoagulants; SGLT2, the sodium/glucose cotransporter 2 inhibitor.

**TABLE 3:** Results of multivariable Cox proportional hazards regression analysis for time to next treatment.

	Univariable			Multivariable		
	HR (95% Cl) *p* value			HR (95% Cl) *p* value		
Age						
≥75	1.06	(0.89–1.25)	0.52			
Sex						
Male	1.02	(0.87–1.21)	0.78			
Complication						
Hypertension	1.10	(0.93–1.29)	0.28			
Dyslipidemia	0.86	(0.70–1.06)	0.16	0.88	(0.62–1.26)	0.50
Diabetes mellitus	1.01	(0.79–1.30)	0.93			
COPD	1.01	(0.74–1.38)	0.93			
Valvular heart disease	0.97	(0.70–1.34)	0.85			
Ischemic heart disease	1.05	(0.89–1.24)	0.55			
Renal impairment	1.31	(1.10–1.57)	<0.003	1.38	(1.15–1.65)	<0.001
Atrial fibrillation and flutter	1.45	(0.96–2.18)	0.075	1.34	(0.88–2.03)	0.17
Concomitant medication						
Statins	0.85	(0.67–1.09)	0.20	0.92	(0.61–1.38)	0.68
β‐Blockers	1.21	(0.82–1.77)	0.33			
NOAC/DOAC	1.11	(0.82–1.50)	0.51			
CCB	1.06	(0.89–1.26)	0.49			
ARB or ACEi	1.05	(0.84–1.30)	0.69			
MRA	1.17	(0.75–1.83)	0.49			
SGLT2	1.47	(0.85–2.56)	0.17	1.62	(1.08–2.42)	0.020
Metformin	1.27	(0.75–2.14)	0.38			
Prior treatment						
Bortezomib	1.10	0.93–1.29	0.28	0.97	0.81–1.16	0.71
Lenalidomide	1.32	1.11–1.56	<0.002	1.38	1.14–1.66	<0.001

Abbreviations: ACEi, angiotensin converting enzyme inhibitor; ARB, angiotensin II receptor blocker; CCB, calcium channel blocker; COPD, chronic obstructive pulmonary disease; DOAC, direct oral anti coagulants; MRA, mineralocorticoid receptor antagonist; NOAC, novel oral anticoagulants; SGLT2, the sodium/glucose cotransporter 2 inhibitor.

**TABLE 4:** Clinical characteristics of carfilzomib treatment group with renal impairment (+)/without renal impairment (−).

	Renal impairment (−)		Renal impairment (+)		
	*n* = 502		*n* = 230		*p*‐value
Characteristic	*n*	%	*n*	%	
Age					
Median, range, (IQR), years	70, 32–90, (63–76)		70, 38–90, (64–77)		0.53
Sex					
Male	261	52	136	59	0.072
Complication					
Hypertension	171	34	101	44	0.011
Dyslipidemia	93	19	45	20	0.74
Diabetes mellitus	60	12	33	14	0.37
COPD	29	6	22	10	0.062
Ischemic heart disease	266	53	141	61	0.036
Valvular heart disease	39	8	18	8	0.98
Atrial fibrillation and flutter	19	4	14	6	0.16
Concomitant medication					
Statins	68	14	32	14	0.89
β‐Blockers	19	4	23	10	<0.001
NOAC/DOAC	46	9	19	8	0.69
CCB	152	30	87	38	0.043
ARB or ACEi	75	15	45	20	0.12
MRA	19	4	10	4	0.72
SGLT2	3	0.6	2	0.9	0.68
Metformin	16	3	8	3	0.84

*Note*: Groups were compared using chi‐square test and Wilcoxon's rank sum test for categorical and continuous variables, respectively.

Abbreviations: ACEi, angiotensin converting enzyme inhibitor; ARB, angiotensin II receptor blocker; CCB, calcium channel blocker; COPD, chronic obstructive pulmonary disease; DOAC, direct oral anti coagulants; MRA, mineralocorticoid receptor antagonist; NOAC, novel oral anticoagulants; SGLT2, the sodium/glucose cotransporter 2 inhibitor.

**TABLE 5:** Total dose of carfilzomib administered for patients in the group with renal impairment (+)/without renal impairment (−).

	Renal impairment (−) *n* = 502	Renal impairment (+) *n* = 230	*p*‐value
Number of administration, Median, Range, (IQR), number	18, 1–170, (8–37)	12, 1–162, (6–29)	0.001
Total accumulated dose, Median, Range, (IQR), mg	985, 30–9380, (420–2230)	760, 30–10 840, (280–1820)	0.003

### 2.4 Statistical analyses

Patient demographics and other disease characteristics were summarized using frequencies and relative frequencies for categorical variables; medians, interquartile ranges, and ranges were computed for continuous variables. Groups were compared using the chi‐square test and Wilcoxon's rank sum test for categorical and continuous variables, respectively. Univariable and multivariable Cox proportional hazards regression analyses were performed to identify the predictors of OS and TTNT. The variables with *p* < 0.20 in the univariable analysis were included in the multivariable analysis. Both OS and TTNT were compared between treatments using the Kaplan–Meier survival analysis, and differences between groups were assessed using the log‐rank test.

All *p*‐values are 2‐sided, and *p* < 0.05 was considered statistically significant. SAS version 9.4 (SAS Institute) was used for all the statistical analyses.

## 3 RESULTS

The data of 732 patients who received carfilzomib treatment for the first time were extracted. Baseline characteristics are listed in Table 1. The median age was 70 years (range 32–90) and 54.2% of the patients were male.

Univariable and multivariable Cox proportional hazards regression analyses predicting OS and TTNT were performed (Tables 2 and 3; Tables S3 and S4). Among baseline characteristics listed as associated with the prognosis of the patients receiving carfilzomib‐based regimens, univariable Cox proportional hazards regression analysis proved that the complication of renal impairment was significantly associated with a higher risk of OS and TTNT with a hazard ratio (HR) of 1.46 (95% confidence interval [CI]: 1.09–1.95; *p* = 0.011) and 1.31 (95% Cl: 1.10–1.57; *p* < 0.003), respectively (Tables 2 and 3). Multivariable Cox regression analysis also showed that only the patients with renal impairment had a significantly higher HR of 1.48 (95% CI: 1.10–2.00; *p* = 0.010) for OS and 1.38 (95% Cl: 1.15–1.65; *p* < 0.001) for TTNT, compared to those without renal impairment (Tables 2 and 3). Univariable Cox proportional hazards regression analysis proved that prior lenalidomide treatment was significantly associated with a higher risk of OS and TTNT with HR of 1.88 (95% Cl: 1.37–2.58; *p* < 0.0001) and 1.32 (95% Cl: 1.11–1.56; *p* < 0.002), respectively (Tables 2 and 3). Multivariable Cox regression analysis also showed that only the patients with prior lenalidomide treatment had a significantly higher HR of 1.80 (95% CI: 1.27–2.55; *p* = 0.0010) for OS and 1.38 (95% Cl: 1.14–1.66; *p* < 0.001) for TTNT, compared to those without prior lenalidomide treatment (Tables 2 and 3). Therefore, renal impairment and prior lenalidomide treatment were independent risk factors regarding OS and TTNT.

### 3.1 Effect of renal impairment

We divided the 732 patients into two groups; 502 patients without renal impairment and 230 patients with renal impairment. The baseline characteristics of the two groups are shown in Table 4. There was no difference in the baseline characteristics between the treatment groups, except in cases with the complication of hypertension (HT), ischemic heart disease (IHD), and concomitant use of β‐blockers (BB) and calcium channel blockers (CCB). The number of cases with the complication of HT, IHD, and concomitant use of BB and CCB among patients with renal impairment was higher than that in patients without renal impairment (HT; 44 vs. 34%, IHD; 61 vs. 53%, BB; 10 vs. 4%, CCB; 38 vs. 30%, respectively). The OS and TTNT were estimated using Kaplan–Meier analysis (Figures 1, 2, 3, 4, 5, 6) (Figures S1 and S2). Patients with renal impairment had significantly inferior OS (log‐rank *p* = 0.011) and TTNT (log‐rank *p* = 0.002) compared to those without renal impairment (Figures 1 and 2). The number of administrations and the total accumulated dose of carfilzomib treatment are listed in Table 5. The number of administrations and total accumulated dose of carfilzomib in patients without renal impairment were higher than those in patients with renal impairment during the follow‐up period (18 vs. 12, 985 mg vs. 760 mg, respectively).

### 3.2 Effect of prior treatment

Patients with prior bortezomib treatment had significantly inferior OS (log‐rank *p* = 0.049) compared to those without prior bortezomib treatment (Figure 3). The two groups had no significant difference in the TTNT (log‐rank *p* = 0.292) (Figure 4). Patients with prior lenalidomide treatment had significantly inferior OS (log‐rank *p* < 0.001) and TTNT (log‐rank *p* = 0.002) compared to those without prior lenalidomide treatment (Figures 5 and 6).

## 4 DISCUSSION AND CONCLUSION

The present study examined the risk factors influencing the OS in patients with RRMM receiving carfilzomib treatment in the real world. To our knowledge, this is the first study to perform a long follow‐up since the approval of carfilzomib in 2014.

Patients with renal impairment had significantly inferior OS and TTNT compared to those without renal impairment. Therefore, a complication of renal impairment was identified as a risk factor regarding OS. Only patients with no or mild renal impairment are generally enrolled in RCTs. However, renal impairment is a common comorbidity, especially in patients with MM, as it is caused by the consequences of myeloma kidney, AL amyloidosis, or chronic kidney disease derived from hypertension or diabetes mellitus. Also, renal impairment might complicate drug dosing and limit treatment options, leading to higher incidence or worsening of adverse events.

Consistent with the present study, the subgroup analysis of the Phase 3 study, ENDEAVOR reported that patients achieving complete renal response had better PFS and OS outcomes than non‐responders across treatment groups.^13^ This study showed that improved renal response is associated with better survival outcomes in patients with baseline renal impairment.

In previous study assessed carfilzomib pharmacokinetics (PK) and pharmacodynamic (PD) in patients with MM and renal impairment, including patients on dialysis, carfilzomib PK was not appreciably altered in patients with renal impairment, including those on dialysis.^14^, ^15^ In addition, PD analysis confirmed prolonged and substantial proteasome inhibition by carfilzomib in patients with renal impairment, and carfilzomib demonstrated in clinically significant responses. Therefore, this study summarized that the dose and treatment schedule of carfilzomib do not need to be adjusted in patients with renal impairment.^14^, ^15^ In other previous study, carfilzomib‐induced deterioration of renal function associated with decreased eGFR was considered transient and dose‐dependent.^4^ Furthermore, this study showed that carfilzomib therapy improved renal function in patients with myeloma‐related renal dysfunction.

The present study showed that the total carfilzomib dose in patients with renal impairment was lower than that in patients without renal impairment. In real‐world settings, physicians might have reduced carfilzomib dose or discontinued administration in patients with renal impairment.

Therefore, physicians might reduce the dose of carfilzomib or discontinue the administration to avoid renal and cardiovascular toxicity in clinical practice. In such cases, the dose might not be sufficient for an effective antitumor effect. Both the number and total accumulated dose of carfilzomib administered were higher in the group of patients without renal impairment than in the group with renal impairment. Therefore, patients with MM having cardiovascular or renal impairment complications might not have received the full dose or regular schedule dosing during carfilzomib‐containing regimens. Therefore, patients without renal impairment showed that favorable OS outcomes compared to those with renal impairment.

Patients with prior lenalidomide treatment had significantly inferior OS and TTNT compared to those without prior lenalidomide treatment. It can be considered that myeloma cells with repeated relapse have become more malignant. Therefore, prior lenalidomide treatment was identified as a risk factor for OS. Consistent with the present study, patients of KRd (carfilzomib, lenalidomide, and dexamethasone) treatment with prior lenalidomide treatment had significantly superior OS and TTNT compared to those without prior lenalidomide treatment.^16^

There was no association between cardiovascular events and TTNT/OS in patients with cardiovascular complications. In the prospective observational study that evaluated the incidence of cardiovascular events in PIs therapy, patients who experienced a cardiovascular event exhibited shorter PFS and OS than those who did not.^2^ Not all patients who exhibited elevated brain natriuretic peptide (BNP) had clinical symptoms or required hospitalization.^2^ Therefore, in the present study, patients might have no clinical symptoms. Although common, cardiovascular events with carfilzomib did not necessarily require dosing discontinuation.^2^ A meta‐analysis evaluating carfilzomib cardiotoxicity reported that high‐dose regimens were associated with cardiotoxicity.^17^ In addition, comorbid hypertension was a major contributor to carfilzomib‐associated cardiovascular events.^17^ In the present study, 37% of the patients exhibited hypertensive complications, 33% received CCBs, and 16% received ACEi or ARB. Therefore, it is considered that our results did not reflect the occurrence of cardiovascular events, as these patients may have been controlled by supportive care. Most clinicians carefully evaluate cardiac function before carfilzomib treatment initiation to determine whether it is appropriate for use. In this context, carfilzomib use did not lead to a significant increase in the number of cardiac‐related events. Patients who are likely to develop heart failure should not receive carfilzomib. Furthermore, BNP and electrocardiograms are used to assess patient cardiac function over time; these findings also relate to the customary clinical management during carfilzomib administration.

The present study has certain limitations. First, this dataset has no clinical test data. Therefore, the severity, stage, and type of MM and renal impairment assessed using blood tests (or clinical examination) cannot be evaluated. Second, the detailed regimens could not be identified from this database. Third, this dataset cannot recognize the prior treatment history of regimens of MM. Thus, a future prospective study is needed to strengthen our conclusions. Despite these, the higher median follow‐up period of 7 years compared to that in the other trials is a significant advantage when interpreting the results and deriving a conclusion.

In conclusion, patients with renal impairment showed significantly inferior OS and TTNT than those without renal impairment. Patients with prior lenalidomide treatment showed significantly inferior OS and TTNT than those without prior treatment. Comorbid renal impairment and prior lenalidomide treatment may be risk factors for predicting unfavorable OS following carfilzomib treatment in a real‐world setting. However, it is necessary to validate these results through a prospective study that includes clinical test data.

## AUTHOR CONTRIBUTIONS

**Hiromi Hagiwara:** Conceptualization (lead); data curation (lead); formal analysis (lead); funding acquisition (lead); investigation (lead); methodology (lead); project administration (lead); resources (equal); software (lead); supervision (lead); validation (lead); visualization (lead); writing – original draft (lead); writing – review and editing (lead). **Takafumi Nakayama:** Methodology (supporting). **Hiroya Hashimoto:** Formal analysis (equal); methodology (supporting); software (supporting); visualization (equal). **Shigeru Kusumoto:** Writing – review and editing (supporting). **Hidekatsu Fukuta:** Methodology (supporting). **Takeshi Kamiya:** Funding acquisition (supporting); resources (supporting). **Koichi Ikuta:** Supervision (equal); writing – review and editing (equal). **Shinsuke Iida:** Conceptualization (lead); methodology (lead); supervision (lead); writing – original draft (supporting); writing – review and editing (lead).

## CONFLICT OF INTEREST STATEMENT

Shinsuke Iida has received honoraria from Sanofi, Janssen, Bristol‐Myers Squibb, Pfizer, Takeda, and Ono Pharmaceutical; and research grants from Chugai, Amgen, AbbVie, GlaxoSmithKline, Daiichi Sankyo, Sanofi, Janssen, Bristol‐Myers Squibb, Pfizer, Takeda, and Ono Pharmaceutical; and is an editorial board member. Shigeru Kusumoto has received research funding from Ono Pharmaceutical Co., Chugai, and Daiichi Sankyo Ltd; and honoraria from Ono, Chugai, and Janssen. Takeshi Kamiya has received honoraria from AstraZeneca K.K., EA Pharma Co., Ltd, Takeda Pharmaceutical Company Limited, TOA Biopharma Co., Ltd, and MIYARISAN Pharmaceutical Co., Ltd. Hiromi Hagiwara, Takafumi Nakayama, Hiroya Hashimoto, Hidekatsu Fukuta and Koichi Ikuta have no conflicts of interest to declare.

## ETHICAL APPROVAL STATEMENT

Not applicable.

## Supporting information


Figure S1.

Figure S2.

Table S1‐S2.

Table S3‐S5.

## Data Availability

The JMDC database is not contractually authorized for public access. Data archiving is not mandated, but data will be made available at reasonable request.
