# Supplementary figures and images for: Risk factors associated with overall survival in patients with multiple myeloma following carfilzomib treatment: A retrospective study from a large claims database in Japan
Source: Cancer Med. 2023 Sep 26;12(19):19361–71. doi: 10.1002/cam4.6457 (PMC10587963; doi:10.1002/cam4.6457)

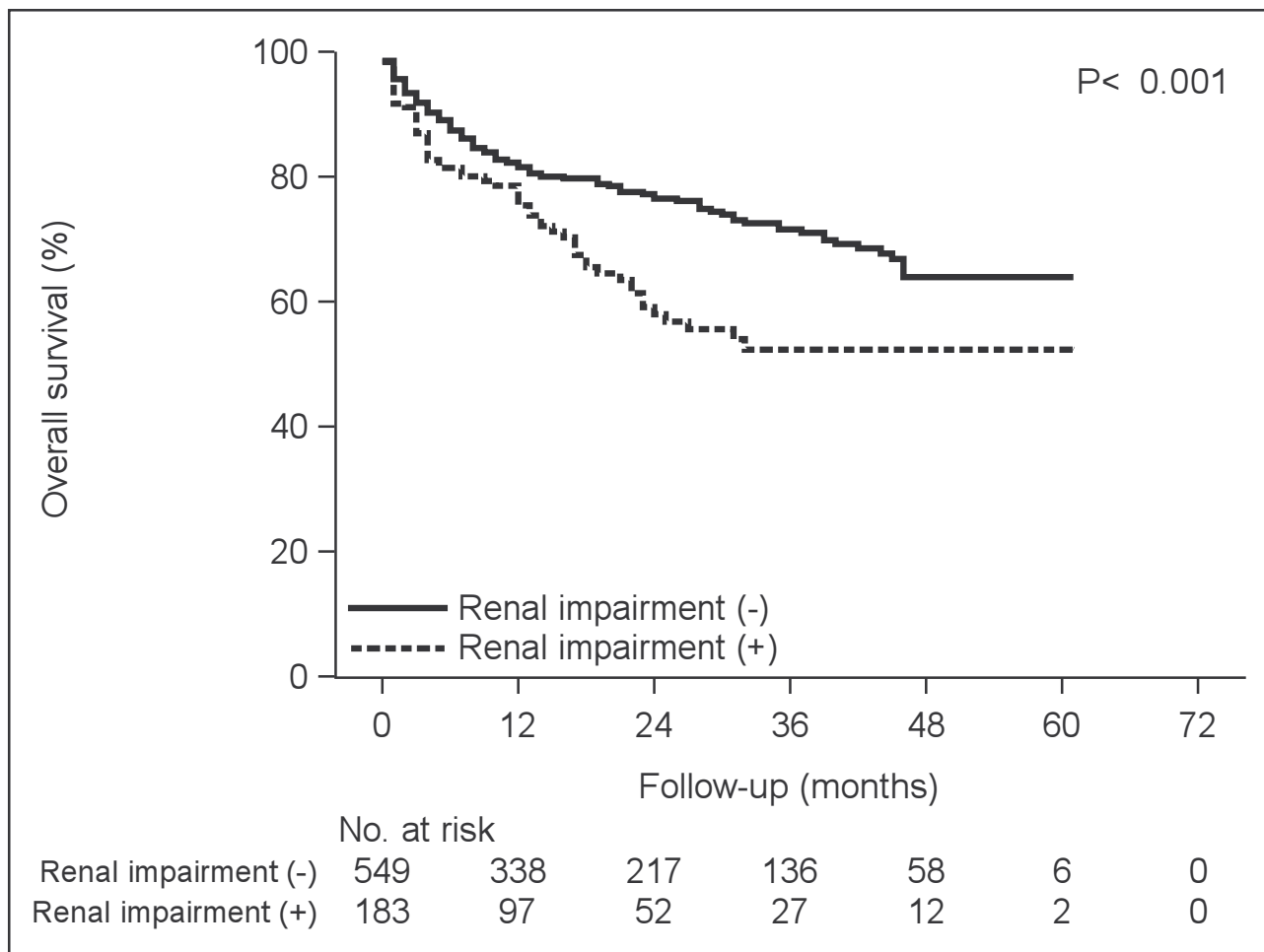

Supplement: Supplementary file 1 — Figure S1. [file CAM4-12-19361-s004.pdf]

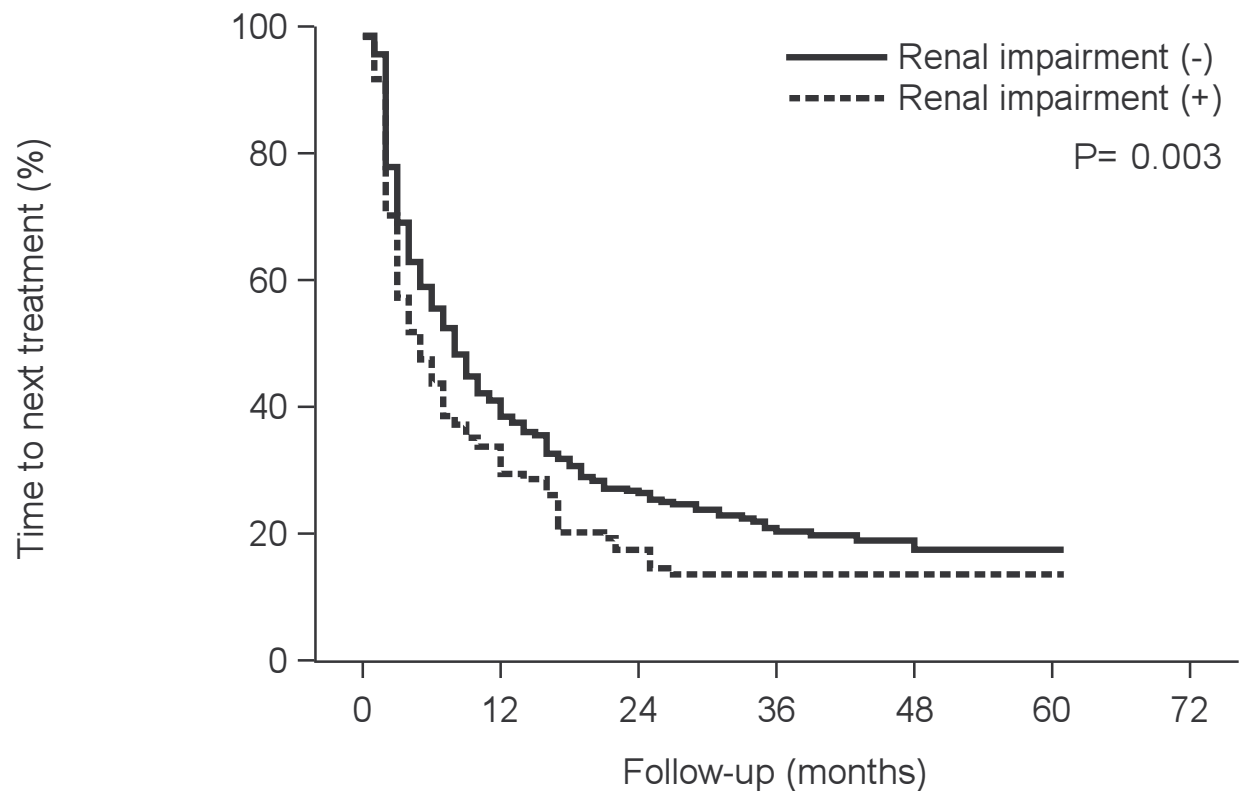

|                      | No. at risk |     |    |    |    |   |   |
|----------------------|-------------|-----|----|----|----|---|---|
| Renal impairment (-) | 549         | 177 | 77 | 38 | 13 | 4 | 0 |
| Renal impairment (+) | 183         | 42  | 17 | 7  | 5  | 2 | 0 |

Supplement: Supplementary file 2 — Figure S2. [file CAM4-12-19361-s003.pdf]
