# Supplementary material for: Risk factors associated with overall survival in patients with multiple myeloma following carfilzomib treatment: A retrospective study from a large claims database in Japan
Source: Cancer Med. 2023 Sep 26;12(19):19361–71. doi: 10.1002/cam4.6457 (PMC10587963; doi:10.1002/cam4.6457)
Supplement: Supplementary file 4 — Table S3‐S5. [file CAM4-12-19361-s001.docx]

Table S3 Results of multivariable Cox proportional hazards regression analysis for overall survival

|  | Univariable | | | Multivariable | | | |
| --- | --- | --- | --- | --- | --- | --- | --- |
|  | HR ( 95 % Cl ) p value | | | HR ( 95 % Cl ) p value | | | |
| **Age** |  |  |  |  |  | |  |
| ≥75y | 1.39 | ( 1.04−1.86 ) | 0.025 | 1.24 | ( 0.92−1.66 ) | 0.16 | |
| **Sex** |  |  |  |  |  |  | |
| Male | 0.98 | ( 0.74−1.29 ) | 0.87 |  |  |  | |
| **Complication** |  |  |  |  |  |  | |
| Hypertension | 1.11 | ( 0.84−1.48 ) | 0.46 |  |  |  | |
| Dyslipidemia | 0.86 | ( 0.60−1.24 ) | 0.41 |  |  |  | |
| Diabetes mellitus | 1.35 | ( 0.92−1.99 ) | 0.12 | 1.26 | ( 0.86−1.84 ) | 0.24 | |
| COPD | 0.94 | ( 0.52−1.70 ) | 0.84 |  |  |  | |
| Valvular heart disease | 1.18 | ( 0.70−2.00 ) | 0.53 |  |  |  | |
| Ischemic heart disease | 1.05 | ( 0.79−1.39 ) | 0.75 |  |  |  | |
| Renal impairment | 1.66 | ( 1.23−2.25 ) | <0.001 | 1.55 | ( 1.13−2.13 ) | <0.007 | |
| Atrial fibrillation and flutter | 1.60 | ( 0.88−2.89 ) | 0.12 | 1.29 | ( 0.71−2.35 ) | 0.40 | |
| **Concomitant medication** |  |  |  |  |  |  | |
| Statins | 0.94 | ( 0.63−1.40 ) | 0.75 |  |  |  | |
| β-blockers | 1.17 | ( 0.66−2.06 ) | 0.59 |  |  |  | |
| NOAC/DOAC | 0.72 | ( 0.43−1.20 ) | 0.20 |  |  |  | |
| CCB | 1.14 | ( 0.85−1.53 ) | 0.37 |  |  |  | |
| ARB or ACEi | 0.93 | ( 0.64−1.36 ) | 0.70 |  |  |  | |
| MRA | 1.53 | ( 0.80−2.91 ) | 0.19 | 1.35 | ( 0.68−2.68 ) | 0.39 | |
| SGLT2 | 0.70 | ( 0.09−5.67 ) | 0.74 |  |  |  | |
| Metformin | 1.14 | ( 0.48−2.73 ) | 0.76 |  |  | |  |
| **Prior treatment** |  |  |  |  |  | |  |
| Bortezomib | 1.35 | ( 1.00−1.81 ) | 0.048 | 1.03 | 0.74-1.43 | | 0.87 |
| Lenalidomide | 1.88 | ( 1.37−2.58 ) | <.0001 | 1.73 | 1.23-2.44 | | <0.002 |

ACEi, angiotensin converting enzyme inhibitor; ARB, angiotensin Ⅱ receptor blocker; CCB, calcium channel blocker; COPD, chronic obstructive pulmonary disease; DOAC, direct oral anti coagulants; MRA, mineralocorticoid receptor antagonist; NOAC, novel oral anticoagulants; SGLT2, the sodium/glucose cotransporter 2 inhibitor.

Table S4 Results of multivariable Cox proportional hazards regression analysis for time to next treatment

|  | Univariable | | | Multivariable | | |
| --- | --- | --- | --- | --- | --- | --- |
|  | HR ( 95 % Cl ) p value | | | HR ( 95 % Cl ) p value | | |
| **Age** |  |  |  |  |  |  |
| ≥75y | 1.06 | ( 0.89−1.25 ) | 0.52 |  |  |  |
| **Sex** |  |  |  |  |  |  |
| Male | 1.02 | ( 0.87−1.20 ) | 0.78 |  |  |  |
| **Complication** |  |  |  |  |  |  |
| Hypertension | 1.10 | ( 0.93−1.29 ) | 0.28 |  |  |  |
| Dyslipidemia | 0.86 | ( 0.70−1.06 ) | 0.16 | 0.87 | ( 0.61−1.25 ) | 0.45 |
| Diabetes mellitus | 1.01 | ( 0.79−1.30 ) | 0.93 |  |  |  |
| COPD | 1.01 | ( 0.74−1.38 ) | 0.93 |  |  |  |
| Valvular heart disease | 0.97 | ( 0.70 – 1.34 ) | 0.85 |  |  |  |
| Ischemic heart disease | 1.05 | ( 0.89−1.24 ) | 0.55 |  |  |  |
| Renal impairment | 1.33 | ( 1.10−1.60 ) | <0.004 | 1.36 | ( 1.12−1.65) | 0.002 |
| Atrial fibrillation and flutter | 1.45 | ( 0.96−2.18 ) | 0.075 | 1.33 | ( 0.88−2.02) | 0.18 |
| **Concomitant medication** |  |  |  |  |  |  |
| Statins | 0.85 | ( 0.67−1.09 ) | 0.20 | 0.93 | ( 0.62−1.41 ) | 0.74 |
| β-blockers | 1.21 | ( 0.82−1.77 ) | 0.33 |  |  |  |
| NOAC/DOAC | 1.11 | ( 0.82−1.50 ) | 0.51 |  |  |  |
| CCB | 1.06 | ( 0.89−1.26 ) | 0.49 |  |  |  |
| ARB or ACEi | 1.05 | ( 0.84−1.30 ) | 0.69 |  |  |  |
| MRA | 1.17 | ( 0.75–1.83 ) | 0.49 |  |  |  |
| SGLT2 | 1.47 | ( 0.85–2.56 ) | 0.17 | 1.73 | ( 1.05−2.85 ) | 0.032 |
| Metformin | 1.27 | ( 0.75−2.14 ) | 0.38 |  |  |  |
| **Prior treatment** |  |  |  |  |  |  |
| Bortezomib | 1.10 | 0.93−1.29 | 0.28 | 0.95 | 0.80-1.14 | 0.60 |
| Lenalidomide | 1.32 | 1.11−1.56 | <0.002 | 1.33 | 1.11-1.60 | 0.002 |

ACEi, angiotensin converting enzyme inhibitor; ARB, angiotensin Ⅱ receptor blocker; CCB, calcium channel blocker; COPD, chronic obstructive pulmonary disease; DOAC, direct oral anti coagulants; MRA, mineralocorticoid receptor antagonist; NOAC, novel oral anticoagulants; SGLT2, the sodium/glucose cotransporter 2 inhibitor.

Table S5 Baseline characteristics of carfilzomib treatment group

| Renal impairment (-)  n | Renal impairment (+)  n | Total  n |
| --- | --- | --- |
| 549 | 183 | 732 |
